# An Automated Diagnosis Method for Lung Cancer Target Detection and Subtype Classification-Based CT Scans

**DOI:** 10.3390/bioengineering11080767

**Published:** 2024-07-30

**Authors:** Lingfei Wang, Chenghao Zhang, Yu Zhang, Jin Li

**Affiliations:** College of Intelligent Systems Science and Engineering, Harbin Engineering University, Harbin 150001, China; wanglingfei@hrbeu.edu.cn (L.W.); 910381219@hrbeu.edu.cn (C.Z.); zhangyu04@hrbeu.edu.cn (Y.Z.)

**Keywords:** lung cancer subtypes classification, lung cancer detection, YOLO V8, attention mechanism

## Abstract

When dealing with small targets in lung cancer detection, the YOLO V8 algorithm may encounter false positives and misses. To address this issue, this study proposes an enhanced YOLO V8 detection model. The model integrates a large separable kernel attention mechanism into the C2f module to expand the information retrieval range, strengthens the extraction of lung cancer features in the Backbone section, and achieves effective interaction between multi-scale features in the Neck section, thereby enhancing feature representation and robustness. Additionally, depth-wise convolution and Coordinate Attention mechanisms are embedded in the Fast Spatial Pyramid Pooling module to reduce feature loss and improve detection accuracy. This study introduces a Minimum Point Distance-based IOU loss to enhance correlation between predicted and ground truth bounding boxes, improving adaptability and accuracy in small target detection. Experimental validation demonstrates that the improved network outperforms other mainstream detection networks in terms of average precision values and surpasses other classification networks in terms of accuracy. These findings validate the outstanding performance of the enhanced model in the localization and recognition aspects of lung cancer auxiliary diagnosis.

## 1. Introduction

Cancer, as a severe malignant tumor, has seen rapidly increasing incidence and mortality rates among the elderly population, posing a significant threat to their health [1]. Smoking, secondhand smoke, air pollution, and high levels of arsenic in drinking water have been identified as major risk factors for cancer, with smoking accounting for the majority of cancer cases. Additionally, factors such as decreased immunity and metabolic changes contribute to the heightened risk of cancer in the elderly. Therefore, the prevention and control of cancer are particularly urgent for the elderly population.

Lung cancer is primarily classified into two types: small cell lung cancer (SCLC) and non-small cell lung cancer (NSCLC), with the latter comprising various subtypes [2]. The major subtypes of NSCLC include large cell carcinoma, adenocarcinoma, and squamous cell carcinoma, each requiring different treatment approaches. These subtypes exhibit variations in both pathology and therapeutic methods. Large cell carcinoma, a subtype of NSCLC, typically grows rapidly and is characterized by large cell morphology, yet it lacks the cytological features of adenocarcinoma or squamous cell carcinoma. Adenocarcinoma, the most common subtype of NSCLC, generally originates in the alveoli, is less associated with smoking, and is more prevalent among non-smokers. Squamous cell carcinoma usually arises from the bronchial epithelium, is closely related to smoking, and commonly occurs in the central part of the lungs.

Medical imaging technology occupies a central role in lung cancer screening, with CT (computed tomography) being widely used as a routine screening method. CT technology provides high-resolution cross-sectional images, thoroughly depicting the anatomical structures of the lungs, thus assisting physicians in accurately identifying pulmonary abnormalities. Despite the significant diagnostic value of CT, its efficacy in diagnosing small lung cancer lesions may be limited, presenting risks of false positives and false negatives. Such potential diagnostic errors can lead to misdiagnoses or missed diagnoses [3,4,5,6]. Moreover, the considerable variation in the size and shape of lung cancer lesions makes small targets in traditional images difficult to recognize with the naked eye, and their class distribution can be extremely imbalanced. The complex anatomical structure and texture of lung images further complicate the distinction between lung cancer nodules and normal structures, requiring automatic detection algorithms to possess high sensitivity and accuracy. These limitations may lead to missed diagnoses of small tumors, restricting their application in diagnostics. Therefore, the development of lung cancer detection and classification models is crucial to better assist radiologists in navigating data and improving diagnostic accuracy.

With the rapid advancement of deep learning technology, deep learning-based methods for lung cancer detection have been widely employed in the auxiliary diagnosis and treatment of lung cancer images obtained from CT scans. These methods utilize convolutional neural networks (CNNs) to conduct in-depth analysis and interpretation of medical images. Despite the significant progress made by CNN-based approaches, they may exhibit lower sensitivity in detecting small lung cancer lesions due to limitations in image resolution or inadequate feature representation. These limitations result in relatively weaker performance in small target detection, sometimes even leading to missed diagnoses or false positives. Therefore, further optimization of deep learning methods to enhance their sensitivity and accuracy in lung cancer detection, particularly for small lesions, is an urgent need in current research.

The challenges of identifying and locating small lesions of lung cancer in medical imaging have prompted the need for detection algorithms. To address this issue, this paper proposes an improved YOLO V8 lung cancer subtype detection model based on the large separable kernel attention mechanism and coordinate attention mechanism. The improvements include deploying a large separable kernel attention, introducing coordinate attention, and using MPDIOU Loss to replace CIOU Loss. These enhancements have yielded positive effects in increasing the model’s focus on lung cancer features, enhancing its perception of small targets, and optimizing target localization capabilities. Based on this, the paper contributes in the following aspects:

(1) The improved method integrates a large separable kernel attention mechanism after the C2f module of the network. This strategy aims to enhance the model’s focus on lung cancer features. By introducing this mechanism into the Backbone section of the C2f layer, the model can more effectively identify and focus on key features in the input images while suppressing the influence of irrelevant features. Applying this attention mechanism in the Neck section of the C2f layer promotes information exchange among features of different scales, further improving the accuracy and robustness of feature representation.
(2) The improved method combines depth-wise convolution with coordinate attention in the SPPF module, aiming to enlarge the model’s receptive field and reduce feature information loss caused by pooling operations. This improvement not only enhances the model’s focus on deep lung cancer features but also improves its perception of lung cancers with different shapes. Consequently, the model can more accurately detect and recognize various subtypes of lung cancer.
(3) The improved method adopts MPDIOU Loss to replace CIOU Loss. To further enhance the accuracy of lung cancer detection and recognition, we introduce MPDIOU Loss to replace the original CIOU Loss. MPDIOU Loss can more accurately measure the differences between predicted boxes and ground truth boxes, thus optimizing the model’s target localization capabilities. By using this new loss function, the model can more accurately calculate IOU loss during the training process and adjust the model parameters accordingly, thereby achieving more precise and efficient detection and recognition of lung cancer subtypes.

## 2. Related Works

### 2.1. The Lung Cancer Detection Method Based on Manually Set Features

The lung cancer detection method based on manually set features primarily focuses on shape analysis. The shape features of lung cancer refer to the geometric shape of its external contour, such as circular or elliptical. Shape analysis involves extracting the boundary of lung cancer and computing the relevant shape measurements. Common methods include morphological operations (such as erosion and dilation) and edge detection. Messay et al. [7] employed a multi-thresholding approach paired with specific morphological operations to better remove unrelated tissues around the nodules. Paing et al. [8] proposed a nodule detection method combining the OTSU algorithm with morphological operations. Gupta et al. [9] first obtained lung masks using the flood fill algorithm and morphological operations, followed by a multi-level thresholding algorithm combined with feature extraction techniques for nodule detection. Soni et al. [10] utilized Sobel and morphological operations for automatic cancer detection. Sairam et al. [11] introduced an edge detection method based on Canny edge detection for lung tumor detection. Rezaie et al. [12] first selected regions of interest containing suspicious nodules using a thresholding method, followed by nodule localization using edge detection algorithms. These methods encompass morphological operations, edge detection, multi-thresholding techniques, and their combinations. However, these methods have some limitations. The manually set features for lung cancer morphological features are susceptible to subjective factors, and these methods may lack robustness in complex situations. Additionally, parameter tuning is required for different cases and datasets, limiting the generality and adaptability of these methods.

### 2.2. Lung Cancer Detection Methods Based on Traditional Machine Learning

Methods based on machine learning are often employed in lung cancer detection tasks using traditional machine learning algorithms, including Support Vector Machines (SVM) [13] and Random Forest [14]. Support Vector Machines are robust supervised learning algorithms widely applied in lung cancer detection tasks. Johora et al. [15] utilized a Gray Level Co-occurrence Matrix (GLCM) to extract features from lung cancer images and subsequently employed Support Vector Machines (SVMs) to distinguish between normal and abnormal lung cells. Qurina et al. [16] also utilized a GLCM to extract corresponding features, followed by the application of Support Vector Machines (SVMs) to differentiate between benign and malignant lung cells. Aghabalaei et al. [17] designed a set of spectral, texture, and shape features to characterize nodules, and then employed SVM classifiers for the classification of suspicious nodules. However, these methods have some limitations. Their reliance on features may lead to insufficient model generalization performance for complex scenarios. Moreover, traditional machine learning methods may struggle to effectively handle high-dimensional, nonlinear medical imaging data, thus limiting their applicability in lung cancer detection.

### 2.3. The Lung Cancer Detection Method Based on Deep Learning

The lung cancer detection methods based on deep learning can be broadly categorized into two main types: single-stage detection and two-stage detection. These two categories of algorithms employ different strategies and network structures for handling lung cancer detection tasks. Lung cancer detection methods based on single-stage detection, such as the YOLO series [18,19,20] or SSD [21], complete the object detection task in a single forward pass stage. For instance, Lin et al. [22] proposed an automatic lesion detection model based on SSD for lung metastases caused by lung cancer in low-resolution SPECT bone scan images. Loraksa et al. [23] proposed the SSD-VGG16 model for detecting nodules of osteosarcoma metastasis to the lungs, enhancing the ability to extract nodules and detection accuracy. Mammeri et al. [24] utilized YOLO V7 for lung nodule detection and introduced a multi-class classification approach. Ji et al. [25] proposed an efficient single-stage ELCT-YOLO model based on improvements to the YOLO V7-tiny model for lung tumor detection in CT images.

Lung cancer detection methods based on two-stage detection, such as the Faster R-CNN [26] and Mask R-CNN [27] series, involve using a Region Proposal Network (RPN) to generate candidate regions and performing detailed classification and position regression on these candidate regions through ROI pooling in two stages. Compared to single-stage methods, they are slightly lagging in real-time performance. Su et al. [28] improved Faster R-CNN from the perspectives of parameter optimization and network model structure, achieving good results in lung cancer detection tasks. Nguyen et al. [29] proposed a new lung nodule detection system based on ResNet architecture to reduce false positives. Xu et al. [30] proposed an improved Faster R-CNN model based on a multi-scale training strategy for lung nodule detection. This model significantly enhances the detection ability for small target nodules by exploiting features from different scale spaces and augmenting low-dimensional features using path augmentation techniques. However, these methods still face challenges, such as poor performance in handling small targets, adaptability to complex backgrounds, and a dependency on large-scale datasets.

## 3. Method

The YOLO V8 algorithm aims to treat the object detection task as a regression problem, directly identifying objects in images through a single neural network model. It divides the input image into grids and predicts the bounding boxes and class probabilities for each grid. Unlike traditional object detection algorithms, YOLO V8 employs multi-scale prediction and Feature Pyramid structures to detect objects of various sizes across different scales. In the YOLO V8 algorithm, the Backbone refers to the backbone network, typically a convolutional neural network (CNN), used to extract features from input images. It is responsible for extracting features from the original images, converting them into feature maps with rich semantic information. The Backbone usually adopts classical CNN architectures such as Darknet, ResNet, or MobileNet to extract features. The Neck section further processes and fuses the feature maps extracted by the Backbone to enhance the model’s perception of targets. It typically includes operations such as Feature Pyramid Network (FPN) and attention mechanisms to fuse feature maps from different levels and enable better understanding of the image’s semantic information. The Head section is responsible for performing the object detection task. It receives the feature maps from the Neck module and generates predictions for the positions and categories of targets. The Head module typically consists of convolutional layers, fully connected layers, and output layers used to predict bounding box positions and category probabilities.

**Figure 1:**
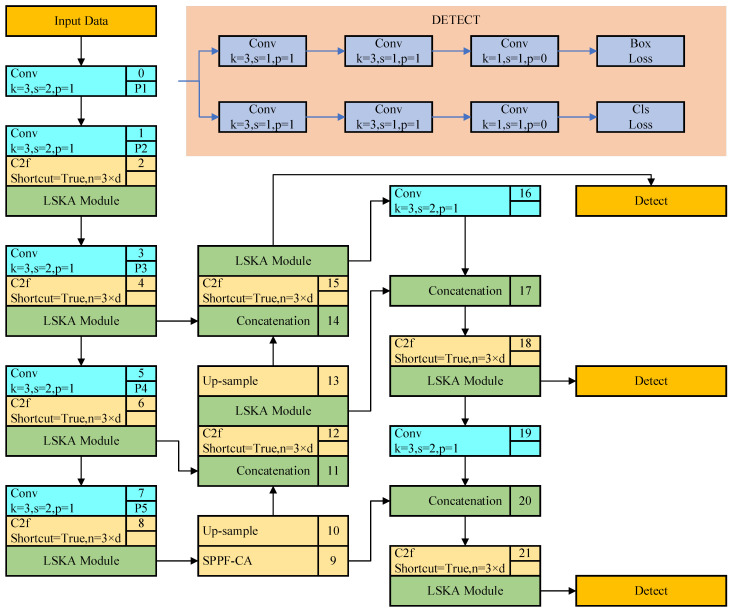
The structure diagram of YOLO V8-LCM.

Despite its advancements, the YOLO V8 algorithm still exhibits limitations in detecting small targets such as lung cancer lesions. This is primarily due to the insensitivity of its Backbone to information regarding small targets. Lung cancer lesions typically occupy a small proportion of the pixels in medical images and exhibit diverse morphologies, making their feature information susceptible to attenuation within the backbone network. Additionally, while the SPPF (Spatial Pyramid Pooling Fast) structure in YOLO V8 helps enhance the model’s adaptability to targets of different scales, its perception ability for small targets like lung cancer lesions remains limited. To address these limitations, this paper proposes an improved YOLO V8 lung cancer subtype detection model. This enhanced model integrates a large separable kernel attention mechanism and a coordinate attention mechanism to better focus on lung cancer features and enhance the perception of small targets. The structure of the model is illustrated in Figure 1.

**Figure 2:**
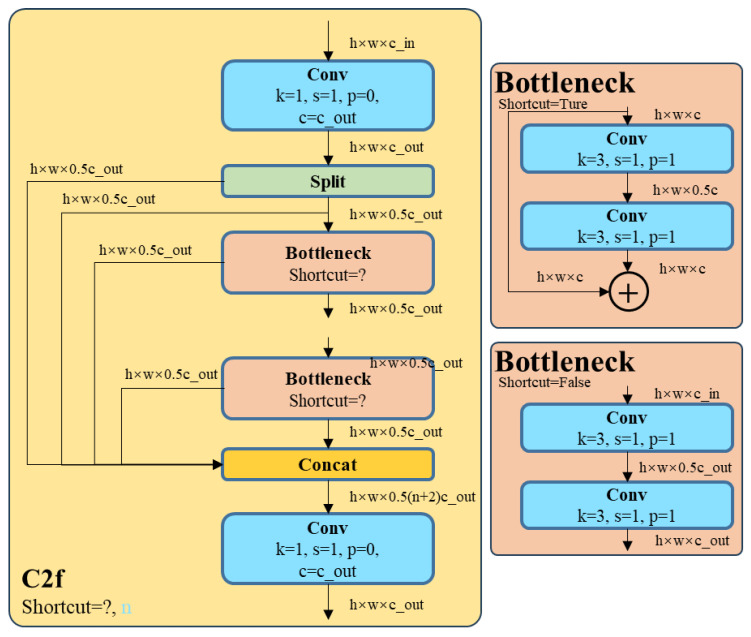
The structure diagram of C2f.

**Figure 3:**
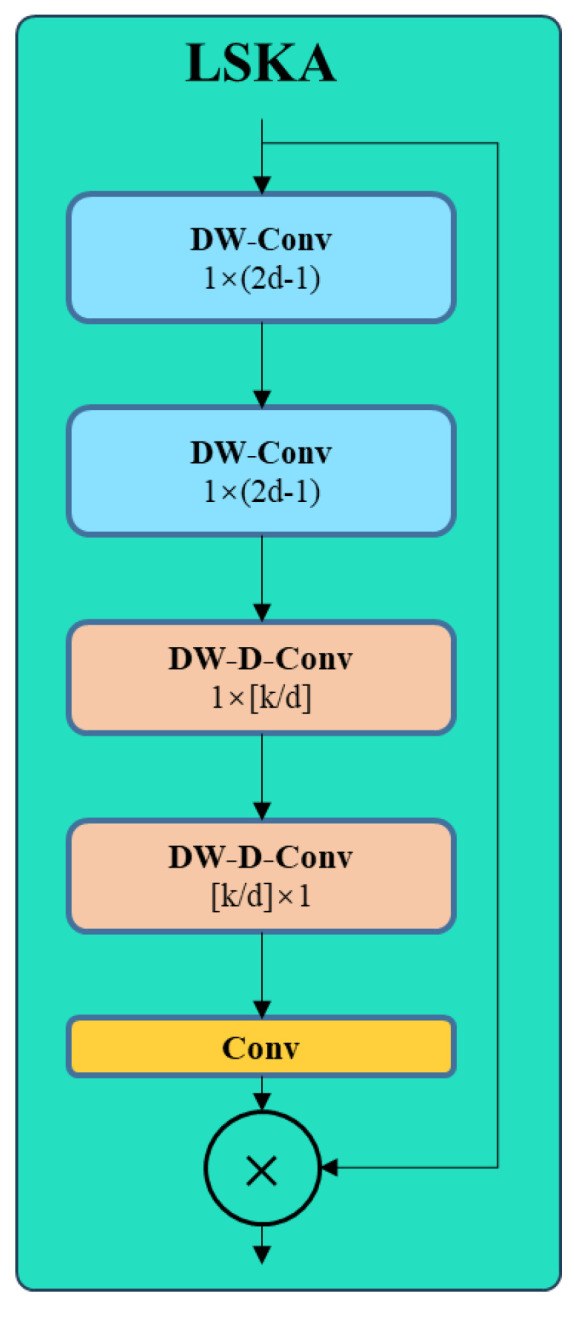
LSKA structure diagram.

Lung cancer medical image data are initially input into the network through the input layer and then proceed to the backbone network, which functions as the feature extraction network. The basic convolution module C2f is shown in Figure 2. Following the C2f module in the backbone network, a large separable kernel attention (LSKA) module is introduced to heighten the model’s attention towards critical regions. The LSKA module is shown in Figure 3. This module amalgamates various convolution types and attention mechanisms to capture key features pertinent to lung cancer more effectively in the image. Subsequently, the feature maps from the backbone network are transmitted to the SPPF-CA module, which focuses on refining the spatial attention region of the model by considering the coordinate relationships between different regions in the image. This facilitates the network in concentrating more deliberately on areas potentially containing lung cancer-related information. To amalgamate the features extracted by different modules, the outputs from the two outputs of the backbone network are directed to the feature fusion module, which also incorporates LSKA. This aids in preserving the vital information extracted by each module to construct a more comprehensive image representation. Finally, the detection head is tasked with generating target boxes, class probabilities, and other pertinent information regarding target detection.

### 3.1. C2f-LSKA Module

In the traditional YOLO V8 architecture, the Backbone and Neck are equipped with C2f modules, as illustrated in Figure 1. The input feature map is defined with dimensions (*n*, *c*, *h*, *w*), where *n* represents the number of Bottlenecks, *c* indicates the number of channels, and *h* and *w* denote the height and width of the feature map, respectively. The feature map (*n*, *c*, *h*, *w*) undergoes splitting through convolutional layers, resulting in two parts: (*n*, 0.5*c*, *h*, *w*). One part directly passes through *n* Bottlenecks, while the other part undergoes a Shortcut operation after each Bottleneck, maintaining a size of (*n*, 0.5*c*, *h*, *w*), before ultimately being output through convolutional layers. In the YOLO V8 object detection framework, the application of C2f in both the Backbone and Neck parts presents evident disadvantages for lung cancer detection. Specifically, within the Backbone, the extensive convolutional layers of C2f lead to the gradual loss of lung cancer feature information as the network deepens. Furthermore, the relatively large down-sampling factor of YOLO V8 further reduces the resolution of the feature map, making it challenging to capture detailed information about lung cancer. In the Neck part, C2f encounters difficulties in feature fusion and smooth information propagation, hindering the effective integration and utilization of feature maps from different layers, thereby compromising the detection performance for small targets. To address these challenges, this paper introduces the large separable kernel attention (LSKA) module after the C2f layer to enhance the network’s feature extraction capabilities and increase sensitivity to lung cancer features. The structure diagram of LSKA is shown in Figure 3.

Large separable kernel attention (LSKA) is proposed based on large kernel attention (LKA). LKA consists primarily of three components: depth-wise convolution, depth-wise separable convolution, and convolution, specifically comprising a (2*d* − 1) × (2*d* − 1) convolutional kernel for depth-wise convolution, a (*k*/*d*) × (*k*/*d*) convolutional kernel with dilation rate *d* for depth-wise separable convolution, and a 1 × 1 convolution. When the kernel size of LKA exceeds 23 × 23, its computational complexity and memory consumption still increase. LSKA decomposes the 2D convolutional kernel of the depth-wise convolution layer into cascaded horizontal 1D and vertical 1D kernels. Specifically, LSKA uniformly decomposes the given *k* × *k* convolutional kernel into 1 × *k* and *k* × 1, which are separable convolutional kernels, concatenating them over the input features. The design of LSKA decomposes the first two layers of LKA into four layers, each consisting of two 1D convolutional layers. LSKA not only retains the advantages of LKA in its long-range dependencies and adaptability to spatial and channel dimensions but also maintains higher accuracy with fewer parameters and computational footprints. To better express the structure of LSKA, the mathematical representation of LSKA is provided here, as shown in Equations (1)–(4):

(1) $${\bar{Z}}^{C}=\sum_{H,W}W_{(2d-1)\times 1}^{C}*(\sum_{H,W}W_{1\times (2d+1)}^{C}*F^{C})$$

(2) ZC=∑H,WW⌊kd⌋×1C∗(∑H,WW1×⌊dk⌋C∗Z¯C)

(3) $$A^{C}=W_{1\times 1}*Z^{C}$$

(4) $${\bar{F}}^{C}=A^{C}⊗F^{C}$$

In the expression of LSKA, ${\bar{Z}}^{C}$ represents the output of depth-wise convolution, where the depth-wise convolution is decomposed into two separable convolutions with kernel sizes of (2d−1)×1 and 1×(2d−1). Similarly, $Z^{C}$ represents the output of depth-wise separable convolution, where the depth-wise separable convolution is decomposed into two separable convolutions with kernel sizes of (1×⌊dk⌋) and (⌊kd⌋×1). $A^{C}$ denotes the output of 1×1 convolution. Finally, ${\bar{F}}^{C}$ is the output of LSKA; it is the element-wise product of the attention map $A^{C}$ and the input feature map $F^{C}$.The introduction of the LSKA module aims to enhance the model’s focus on important regions. This module combines various convolutional types and attention mechanisms to more effectively capture critical features related to lung cancer in the image and facilitate feature interaction.

### 3.2. SPPF-CA Module

**Figure 4:**
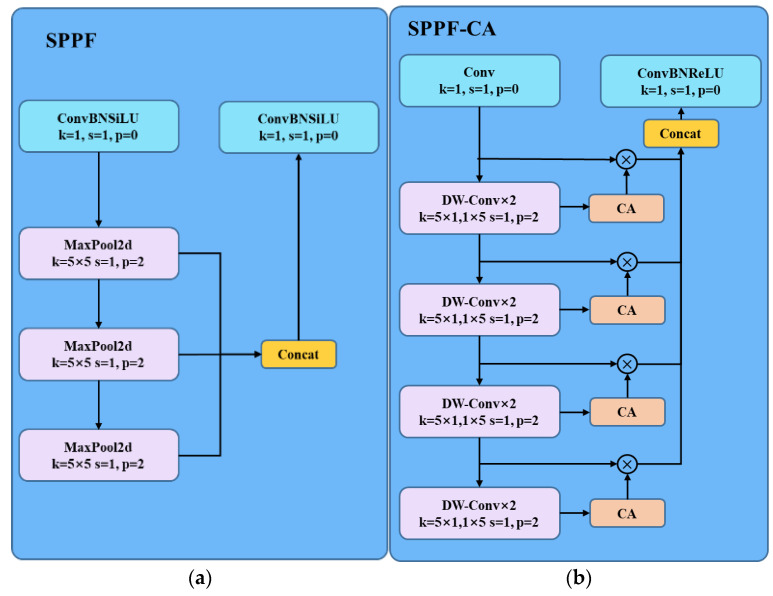
Structure comparison before and after SPPF improvement.

The traditional SPPF structure is illustrated in Figure 4a. Setting the feature map dimension as *N*, after passing through three different Max-Pool layers, it forms different feature vectors of dimensions (1 × 1 × *N*), (5 × 5 × *N*), (9 × 9 × *N*), and (13 × 13 × *N*), which are then concatenated. However, the multi-layer pooling operations in SPPF reduce the spatial resolution of features, potentially leading to the loss of feature information for small targets. Therefore, based on the SPPF structure, we propose a new SPPF-CA structure, as depicted in Figure 4b. Firstly, we introduce deep convolution to flexibly retain more local information through pointwise convolution stages, helping to reduce potential information loss in pooling operations. Additionally, this enhances the receptive field, enabling the model to better understand the global structure of the input image. After the feature map passes through DW-Conv calculation at each layer, it goes through the CA module, followed by concatenation with the input from the previous layer to obtain an output. Finally, the feature maps from the four layers are concatenated to obtain the final output. The CA module can better preserve lung cancer information in deep features, further enhancing the Backbone’s feature extraction capability.

**Figure 5:**
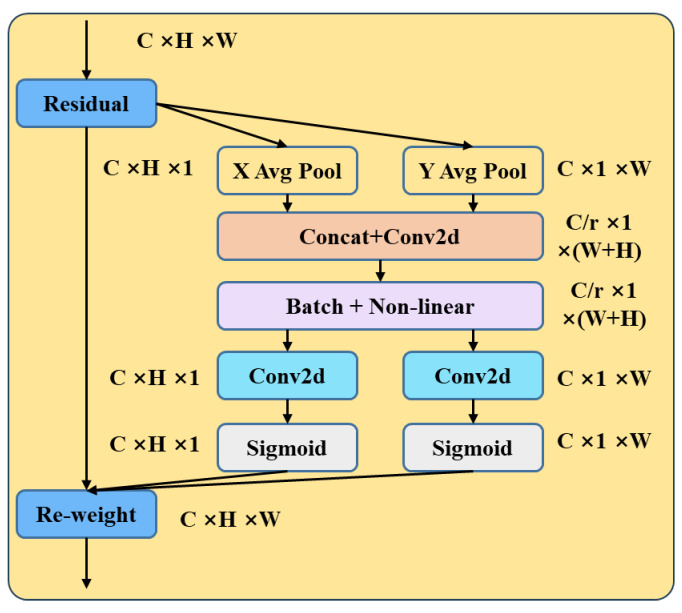
Coordinate Attention structure diagram.

Coordinate Attention (CA) is a type of coordinate attention mechanism designed to encode precise positional information into neural networks for modeling channel relationships and long-term dependencies. This mechanism primarily consists of two steps: Coordinate information embedding and Coordinate Attention generation, as depicted in Figure 5.

In the Coordinate information embedding step, positional information is embedded into the input feature map. This can be achieved by performing global average pooling on the input feature map, dividing it into width and height directions for pooling to obtain feature maps in both width and height directions. Specifically, given the input *X*, we use two spatial range pooling kernels (*H*, 1) or (1, *W*) to encode each channel along the horizontal and vertical coordinates, respectively. Therefore, the formula for the output of channel *c* in height of *h* is as follows:

(5) $$z_{c}^{h}(h)=\frac{1}{W}\sum_{0\leq i\leq W}x_{c}(h,j)$$

Similarly, the output of the *c*-th channel with a width of *w* can be expressed as

(6) $$z_{c}^{w}(w)=\frac{1}{H}\sum_{0\leq i\leq H}x_{c}(j,w)$$

In the Coordinate Attention generation step, attention maps are generated using positional information. Firstly, the feature maps obtained from the width and height directions of the global receptive field are concatenated together. Subsequently, they are fed into a shared convolutional module with a 1 × 1 convolution kernel to reduce their dimensions to the original C/r. Then, the batch-normalized feature maps, denoted as $F_{1}$, are passed through a Sigmoid activation function to obtain feature maps f, denoted as $1\times \left(W+H\right)\times C/r$, given by Equation (7) as follows:

(7) $$f=\delta (F_{1}(\left[z^{h},z^{w}\right]))$$

Here, $\left[z^{h},\cdot z^{w}\right]$ represents the concatenation operation along the spatial dimension, δ is a non-linear activation function, and $f\in R^{C/r\times (H+W)}$ is the intermediate feature map encoding spatial information along the horizontal and vertical directions. r controls the reduction rate of the block size.

Then, the feature map f is convolved separately along the original height and width dimensions with 1 × 1 convolutional kernels to obtain feature maps $F_{h}$ and $F_{w}$ with the same number of channels as the original. After passing through the Sigmoid activation function, attention weights $g^{h}$ for the height and $g^{w}$ for the width are obtained, as expressed in Equations (8) and (9) below:

(8) $$g^{h}=\sigma (F_{h}(f^{h}))$$

(9) $$g^{w}=\sigma (F_{w}(f^{w}))$$

Finally, the feature map with attention weights in both width and height directions is obtained by element-wise multiplication on the original feature map. The expression is as follows:

(10) $$y_{c}(i,j)=x_{c}(i,j)\times g_{c}^{h}(i)\times g_{c}^{w}(j)$$

The advantage of the CA attention mechanism lies in its ability to participate in modeling large regions while avoiding significant computational overhead. It considers not only channel information but also directionally relevant positional information, making it sufficiently flexible and lightweight. It can be easily integrated into the core modules of lightweight networks.

The design of the Feature Pyramid (SPPF) in YOLO V8 is primarily intended to extract feature information from different scales. However, for lung cancer, its feature information may be sparse and complex, and the SPPF may not effectively extract its features. Additionally, the pooling operations in the SPPF may lead to the loss of lung cancer information. The SPPF-CA structure proposed in this paper replaces the MaxPool2d layer with deep convolution and integrates Coordinate Attention (CA) to enhance the capture of features of different sizes, promoting better integration of lung cancer features across various scales.

Assuming the input features to the SPPF-CA module are denoted by $f_{in}^{C}$, the specific mathematical process of the SPPF-CA module can be described as follows:

(11) $$f_{in1}^{C}=W_{1\times 1}^{C}*f_{in}^{C}$$

(12) $$f_{in2}^{C}=\sum_{H,W}W_{5\times 1}^{C}*(\sum_{H,W}W_{1\times 5}^{C}*f_{1}^{C})$$

(13) $$f_{in-n}^{C}=\sum_{H,W}W_{5\times 1}^{C}*(\sum_{H,W}W_{1\times 5}^{C}*f_{in-(n-1)}^{C})$$

(14) $$f_{out-n}^{C}=f_{in-n}^{C}⊗CA(f_{in-(n+1)}^{C})$$

(15) $$f_{out}^{C}=W_{1\times 1}^{C}*concat(f_{out1}^{C},\ldots f_{out-n}^{C})$$

where $f_{in-n}^{C}$ represents the features extracted by depth-wise separable convolution, $f_{out-n}^{C}$ represents the features fused with CA attention, $f_{out}^{C}$ represents the features output by the SPPF-CA module, and CA() denotes the CA attention mechanism described earlier.

### 3.3. MPDIOU Loss

MPDIOU (Minimum Point Distance-based IOU) is a loss function designed for bounding box regression, aimed at addressing the challenge of ineffective optimization when predicted and ground truth bounding boxes have the same aspect ratio but vastly different width and height values. This loss function incorporates three key factors, calculating IOU by minimizing the Point Distance between predicted and ground truth bounding boxes, thereby simplifying the computation process:

(1) Overlap or Non-Overlap Region: Considers the overlap or non-overlap region between two bounding boxes to ensure the loss function responds appropriately to different scenarios.
(2) Center Point Distance: Considers the distance between the center points of predicted and ground truth bounding boxes. By focusing on the position of the center points, MPDIOU can more accurately measure the relative positional relationship between the two bounding boxes.
(3) Width and Height Deviation: Considers the deviation between the width and height of predicted bounding boxes and those of ground truth bounding boxes. This is necessary because, even if the aspect ratios are the same, the actual width and height values may differ, requiring correction for this deviation.

MPDIOU synthesizes the above factors to more accurately measure the similarity between predicted and ground truth bounding boxes, thereby assisting in optimizing the bounding box regression task. The advantage of this approach lies in its ability to effectively handle situations where aspect ratios are the same but width and height values differ, thus improving the performance of bounding box regression.

Suppose at the input end, $B_{prd}=(X_{1}^{prd},Y_{1}^{prd},X_{2}^{prd},Y_{2}^{prd})$ represents the coordinates of the predicted bounding box and $B_{gt}=(X_{1}^{gt},Y_{1}^{gt},X_{2}^{gt},Y_{2}^{gt})$ represents the coordinates of the ground truth bounding box. The width and height of the input image are denoted by *w* and *h*, respectively. $L_{MPDIOU}$ represents the coordinates of the MDPIOU Loss output.

The area of $B_{gt}$ is represented by Formula (16):

(16) $$A^{gt}=(X_{2}^{gt}-X_{1}^{gt})*(Y_{2}^{gt}-Y_{1}^{gt})$$

The area of $B_{prd}$ is represented by Formula (17):

(17) $$A^{prd}=(X_{2}^{prd}-X_{1}^{prd})*(Y_{2}^{prd}-Y_{1}^{prd})$$

The intersection *I* between $B_{prd}$ and $B_{gt}$ is illustrated by Formulas (18)–(20):

(18) $$X_{1}^{I}=\max(X_{1}^{prd},X_{1}^{gt}),X_{2}^{I}=\min(X_{2}^{prd},X_{2}^{gt})$$

(19) $$Y_{1}^{I}=\max(Y_{1}^{prd},Y_{1}^{gt}),Y_{2}^{I}=\min(Y_{2}^{prd},Y_{2}^{gt})$$

(20) $$I=\left\{\begin{array}{ll} \left(X_{2}^{I}-X_{1}^{I})*(Y_{2}^{I}-Y_{1}^{I}\right) & ,\;if\;X_{2}^{I}>X_{1}^{I},Y_{2}^{I}>Y_{1}^{I}\\ 0 & ,\;otherwise \end{array}\right.$$

The calculation method for the MPDIOU Loss is illustrated by Formulas (21)–(26):

(21) $$d_{1}^{2}={\left(X_{1}^{prd}-X_{1}^{gt}\right)}^{2}+{\left(Y_{1}^{prd}-Y_{1}^{gt}\right)}^{2}$$

(22) $$d_{2}^{2}={\left(X_{2}^{prd}-X_{2}^{gt}\right)}^{2}+{\left(Y_{2}^{prd}-Y_{2}^{gt}\right)}^{2}$$

(23) $$u=A^{gt}+A^{prd}-I$$

(24) IOU=Iu

(25) $$MPDIOU=IOU-\frac{d_{1}^{2}}{h^{2}+w^{2}}-\frac{d_{2}^{2}}{h^{2}+w^{2}}$$

(26) $$L_{IOU}=1-IOU,\;L_{MPDIOU}=1-MPDIOU$$

### 3.4. Evaluation Metrics

To evaluate the performance of models in lung cancer detection tasks, this study selected metrics such as precision, recall, and accuracy. Specifically, TP refers to the number of instances where the model predicts the positive class correctly; FP refers to the number of instances where the model predicts the positive class incorrectly; FN refers to the number of instances where the model predicts the negative class incorrectly; and TN refers to the number of instances where the model predicts the negative class correctly.

(1) Precision: Precision, also known as positive predictive value, refers to the proportion of instances predicted as positive by the model that are actually positive. A high precision indicates that the model’s predictions of the positive class are more accurate. Precision is defined by Formula (27):

(27) $$P=\frac{TP}{TP+FP}$$

(2) Recall: Recall, also known as sensitivity or true positive rate, is defined as the proportion of true positive instances to all actual positive instances. A high recall indicates that the model can detect positive samples comprehensively. Recall is defined by Formula (28):

(28) $$R=\frac{TP}{TP+FN}$$

(3) Accuracy: Accuracy is defined as the proportion of correctly predicted samples to the total number of samples. A high accuracy indicates that the model can classify samples more accurately. Accuracy is defined by Formula (29):

(29) $$ACC=\frac{TP+TN}{TP+TN+FN+FP}$$

(4) Specificity: Specificity, also known as sensitivity or true negative rate, is typically refers to the identification accuracy of a test or diagnostic method for a specific disease or biological marker. Accuracy is defined by Formula (30):

(30) $$S=\frac{TN}{TN+FP}$$

In addition, this study also employs the common metric in the field of object detection: Mean Average Precision (mAP). In object detection tasks, the calculation of mAP involves computing the average precision for different categories and averaging these average precisions to obtain the final mAP value.

The calculation method for average precision can be represented by Formula (31):

(31) $$AP=\int_{0}^{1}P(R)d_{R}$$

where precision (recall) represents the points on the precision-recall curve calculated based on different confidence threshold values.

The *mAP* is the average of the *AP* values for all classes, calculated as shown in Equation (32):

(32) $$mAP=(\sum_{i=1}^{N}AP_{i})/N$$

where *N* represents the total number of classes. This formula represents the average performance of the model across multiple classes.

In the task of lung cancer detection, this paper selects mAP (50%) and mAP (50–95%) as evaluation metrics, which respectively represent the average precision at different thresholds. mAP (50%) indicates the average precision at an IOU of 50%, while mAP (50–95%) represents the average precision across IOUs ranging from 50% to 95%. These two metrics provide evaluations of the model’s performance at different levels of overlap.

## 4. Experimentation

### 4.1. Data Preprocessing

To enrich the experimental data, we have integrated CT images from Lung-PET-CT-Dx, NSCLC-Radiomics, and NSCLC Radiogenomics to create a dataset comprising four subtypes of lung cancer. The descriptions of each dataset are as follows:

The Lung-PET-CT-Dx dataset is a publicly available dataset focused on lung cancer target detection research. It includes CT, PET, and combined PET/CT volumetric data from 355 lung cancer patients, but the experimental dataset excludes the PET-CT data. Based on histopathological diagnosis, these patients are clearly classified into four major categories: adenocarcinoma, small cell carcinoma, large cell carcinoma, and squamous cell carcinoma. Among them, adenocarcinoma accounts for 265 cases, small cell carcinoma for 44 cases, large cell carcinoma for 5 cases, and squamous cell carcinoma for 62 cases. The tumor locations in the dataset are annotated by five experienced thoracic radiologists, ensuring the accuracy and reliability of the annotations.

The NSCLC-Radiomics dataset is a collection of images from non-small cell lung cancer (NSCLC) patients, comprising data from 422 patients. These cases are subdivided into three categories based on pathological types: adenocarcinoma, large cell carcinoma, and squamous cell carcinoma. Specifically, there are 52 cases of adenocarcinoma, 114 cases of large cell carcinoma, and 152 cases of squamous cell carcinoma, totaling 318 cases. However, 104 cases were excluded from the experimental sample due to an inability to identify the type of cancer. For the remaining patients, radiological oncologists manually delineated the total tumor volume in the CT scan data and provided associated clinical outcome data. The cancerous regions were annotated with rectangular bounding boxes by experts.

The NSCLC-Radiogenomics dataset focuses on radiogenomic research of non-small cell lung cancer and includes data from 211 subjects. This dataset provides computed tomography and positron emission tomography/computed tomography images, but the PET-CT data are excluded from the experimental dataset. The cases are clearly categorized into two types: adenocarcinoma and squamous cell carcinoma. Specifically, there are 112 cases of adenocarcinoma and 29 cases of squamous cell carcinoma. Notably, 3 cases were excluded from the experimental analysis due to an inability to identify the cancerous regions. The tumor locations were annotated by thoracic radiologists, ensuring the accuracy and reliability of the annotations. The cancerous regions were also delineated with rectangular bounding boxes by experts.

We compiled four types of lung cancer diseases from three datasets, including 429 cases of adenocarcinoma, 44 cases of small cell carcinoma, 119 cases of large cell carcinoma, and 243 cases of squamous cell carcinoma. From each adenocarcinoma case, 3 2D images were selected as samples, totaling 1287 2D images. From each small cell carcinoma case, 10 2D images were selected as samples, totaling 440 2D images. From each large cell carcinoma case, 10 2D images were selected as samples, totaling 1190 2D images. From each squamous cell carcinoma case, 5 2D images were selected as samples, totaling 1215 2D images. However, due to the small number of small cell carcinoma samples, the sample data for small cell carcinoma were augmented to 880 images through rotation and translation.

The data preprocessing procedure is as follows: First, denote a point (*x*, *y*) on the original image, which after rotation becomes (*x*′, *y*′). The rotation is centered at (*cx*, *cy*) with an angle of rotation denoted by *θ* (in radians). The rotation method is described by Formulas (33) and (34) as follows:

(33) $$x\prime =\left(x-cx\right)*\cos\left(\theta \right)-\left(y-cy\right)*\sin\left(\theta \right)+cx$$

(34) $$y\prime =\left(x-cx\right)*\sin\left(\theta \right)+\left(y-cy\right)*\cos\left(\theta \right)+cy$$

where *cos* and *sin* represent trigonometric functions used to compute the cosine and sine values, respectively.

The formula for the offset of the point (*x*, *y*) after the displacement by (*dx*, *dy*) is as follows:

(35) x′=x+dx

(36) y′=y+dy

These formulas represent the new coordinates (*x*′, *y*′) after the offset.

The dataset was divided into training, validation, and test sets in an 8:1:1 ratio, and the YOLO V8 framework was trained with default training parameters for 100 epochs, while conducting five-fold cross-validation. The experimental platform software includes the Windows 11 operating system, Python 3.7 programming language, and PyTorch 1.2 software, while the hardware platform consists of an Intel i5-12600K CPU, 16 GB DDR4 4000 MHz memory, and a single Nvidia 2080Ti 11GB VRAM GPU. The pseudocode of the training process is shown in Table 1. The experiments underwent 5-fold cross-validation and the average and standard deviation were calculated.

### 4.2. Sensitivity Analysis

Sensitivity experiments were conducted on the dataset to verify the impact of different components of the improved YOLO V8 on the performance of lung cancer type detection. The accuracy experimental results are presented in Table 2. ALL-LESIONS represents the detection of all types of lung cancer, while Class 0, Class 1, Class 2, and Class 3 respectively represent the classification of small cell carcinoma, large cell carcinoma, adenocarcinoma, and squamous cell carcinoma. P denotes precision, R denotes recall, mAP (50%) represents the mean average precision at an IOU threshold of 50%, and mAP (50–95%) represents the mean average precision within the IOU threshold range of 50% to 95%.

The results of the ablation experiments are shown in Table 3. In the table, ‘L’ represents the large separable kernel attention mechanism, ‘C’ represents the Coordinate Attention mechanism, ‘M’ represents the MPDIOU Loss, and ‘YOLO V8-LCM’ represents the improved YOLO V8 network model. To evaluate the effectiveness of each module, we progressively ablated each module.

To assess the effectiveness of the large separable kernel attention mechanism in the model, we removed the LSKA module and compared it with YOLO V8-LCM. The experimental results show that YOLO V8-LCM outperforms the model without the LSKA module in metrics such as Precision, Recall, mAP (50%), and mAP (50–95%). Specifically, mAP (50%) and mAP (50–95%) decreased by 0.9% and 1.8%, respectively, indicating that the large separable kernel attention plays a crucial role in promoting the lung cancer detection task.

To evaluate the impact of the Coordinate Attention mechanism on model performance, we removed the CA module and compared it with YOLO V8-LCM. The experimental data indicate that the performance of the model decreases after removing the CA module in metrics such as Precision, Recall, mAP (50%), and mAP (50–95%). Particularly, mAP (50%) and mAP (50–95%) decreased by 0.6% and 1.6%, respectively, demonstrating that the Coordinate Attention mechanism has a positive impact on the accuracy and discriminability of detection results.

To assess the effectiveness of MPDIOU Loss, we removed the MPDIOU Loss module and compared it with YOLO V8-LCM. The experimental results show that the performance of the model decreases after removing the different modules in metrics such as Precision, Recall, mAP (50%), and mAP (50–95%). Specifically, mAP (50%) and mAP (50–95%) decreased by 1.1% and 1.52%, respectively, indicating the positive impact of MPDIOU Loss on the lung cancer detection task.

Through the ablation experiments, we validated the critical role of large separable kernel attention, Coordinate Attention, and MPDIOU Loss in improving lung cancer detection performance. Compared to the base model YOLO V8, YOLO V8-CM, YOLO V8-LM, and YOLO V8-LC integrated with these modules exhibited superior performance in all evaluation metrics, demonstrating the effectiveness of each module combination. YOLO V8-LCM stood out among all comparative network structures, achieving significant performance improvements not only in overall lung cancer detection but also in the detection of various types of lung cancer. These results not only confirm the importance of each component in the model but also reveal the mutually reinforcing relationships among the three structures, collectively building an efficient and accurate lung cancer detection network.

### 4.3. Detection Comparative Experiment

This paper introduces a novel object detection model, YOLO V8-LCM, which incorporates an improved attention mechanism (LCM module) on top of YOLO V8. To assess the performance of the new model, a comprehensive comparison was conducted against several classical object detection models. The performance in the test dataset is presented in Table 4 below.

Compared to the classical network architectures mentioned, YOLO V8-LCM demonstrates significant improvements in Precision, Recall, mAP (50%), and mAP (50–95%), showcasing outstanding performance. Particularly, we observed substantial performance gains for YOLO V8-LCM compared to SSD, Faster R-CNN, YOLO V7, and YOLO V3 across performance metrics. This indicates that the proposed model in this paper is a more advanced and effective object detection model.

In the object detection networks listed in Table 4, the feature extraction part often involves extensive pooling and down-sampling operations. This poses a challenge in lung cancer CT image detection, as lung cancer often appears in the form of small targets. These operations can lead to the loss of features related to small targets, thereby reducing detection performance. To address this issue, we propose the YOLO V8-LCM network, which improves performance through the following three enhancements: Firstly, introducing the LSKA module after all C2f layers enhances attention to lung cancer information, facilitating feature extraction and interaction. Secondly, employing the CA module and depth convolutions in the SPPF layer enlarges the receptive field and preserves lung cancer features in deep features, further enhancing the network’s feature extraction capability. Lastly, adopting MPDIOU Loss in the regression branch improves detection accuracy. These improvements collectively enhance YOLO V8-LCM’s ability to detect small targets.

### 4.4. Accuracy Comparison Experiment

This paper conducts a detailed comparison of the proposed method with the latest methods in the field of lung cancer classification. The specific comparison results in references [31,32,33,34,35,36,37,38,39,40] are presented in Table 5. The first row of the table lists key information such as the data modality, dataset source, lung cancer subtypes, sample size, algorithm models, and final accuracy. Analysis of the literature reveals that most lung cancer classification studies are primarily based on CT images. The research results indicate that YOLO V8-LCM demonstrates excellent detection performance for various subtypes of lung cancer on CT data, with an accuracy of 0.961, representing a significant performance improvement compared to other methods. Compared to other algorithms based on the PET/CT data modality, such as ResNet (ACC = 0.81), MobiNet (ACC = 0.74), and Xception (ACC = 0.78), YOLO V8-LCM achieves higher accuracy, demonstrating superior performance. Compared with literature [36], the proposed method is similar to its ACC, but literature [36] uses the DETR method, which only detects three lung cancer subtypes of a single PET/CT modality. Compared with the convolutional neural network, DETR. The method is based on a Transformer, whose model complexity is higher, and the training is more complicated. In comparison to traditional methods like SVM and GBRT, YOLO V8-LCM performs better in terms of accuracy, highlighting the superiority of deep learning models. The model proposed in this paper achieves high accuracy and successfully accomplishes accurate discrimination of lung cancer histological classifications.

### 4.5. Experimental Results Visualization

**Figure 6:**
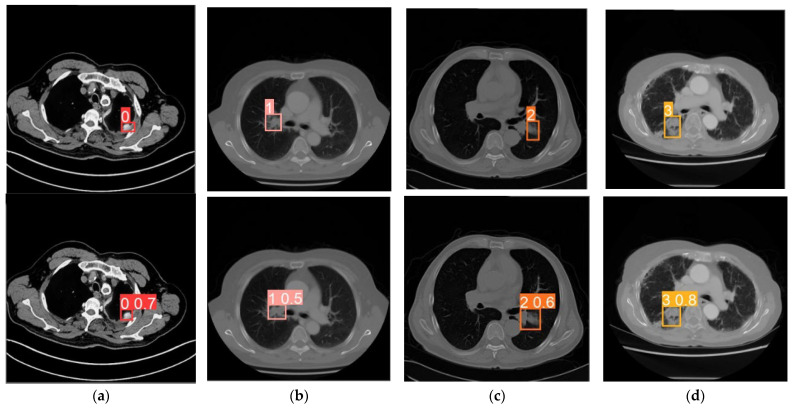
Experimental result diagram. Labels 0, 1, 2, and 3 represent subtypes, with detection targets within the box range and numerical values indicating probabilities. (**a**) Small cell carcinoma. (**b**) Adenocarcinoma. (**c**) Squamous cell carcinoma. (**d**) Large cell carcinoma.

The images in the first row of Figure 6 represent the ground truth, which is the diagnostic result carefully analyzed by medical experts and serves as the benchmark for evaluating the accuracy of the algorithm. The images in the second row depict the predicted results obtained after processing the original images using the algorithm proposed in this paper. This figure demonstrates how the algorithm identifies and analyzes key features in lung cancer images to make predictions.

Figure 6 provides examples of predictions for small cell carcinoma, adenocarcinoma, squamous cell carcinoma, and large cell carcinoma. From the corresponding predicted results, the predicted boxes for lung cancer overlap significantly with the manually annotated boxes, demonstrating that the proposed model accurately locates the lesions of lung cancer. Additionally, the model shows good performance in detecting small targets. The excellent performance of the proposed model relies on specific attention mechanisms and improvements in loss functions, including deploying Coordinate Attention mechanisms in the C2f stage, setting up large separable attention mechanisms in SPPF, and utilizing MPDIOU Loss instead of CIOU Loss.

The images in the first row of Figure 6 represent the original scan images of lung cancer, presenting the raw state of the lungs. The images in the second row depict the visualization results obtained by overlaying the heatmap onto the original images. In these results, different colors in the heatmap indicate different confidence levels predicted by the algorithm, where darker colors indicate higher confidence in the algorithm’s prediction for that region.

**Figure 7:**
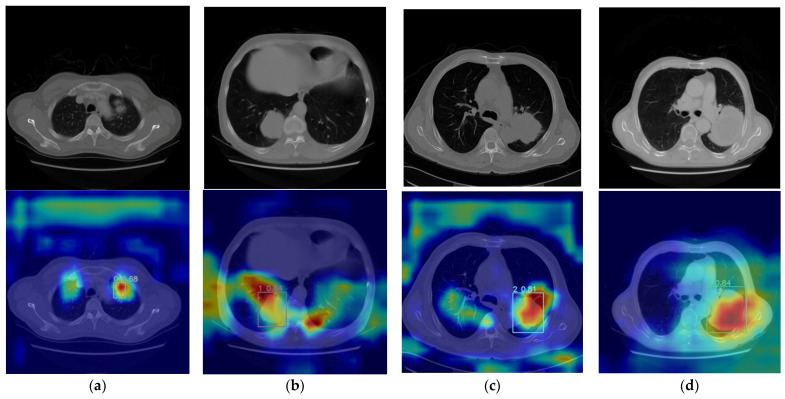
Heatmap visualization. Labels 0, 1, 2, and 3 represent subtypes, with detection targets within the box range and numerical values indicating probabilities. (**a**) Small cell carcinoma. (**b**) Adenocarcinoma. (**c**) Squamous cell carcinoma. (**d**) Large cell carcinoma.

In Figure 7, various subtypes of lung cancer are selected, including small cell carcinoma, adenocarcinoma, squamous cell carcinoma, and large cell carcinoma, and the visualization results for the different diseases are provided. Except for squamous cell carcinoma, the model demonstrates good detection performance for small cell carcinoma, adenocarcinoma, and large cell carcinoma. The visualization results of lung cancer detection accurately showcase the position, morphology, and category recognition ability of lung cancer lesions, providing a certain level of interpretability. These visualization results further validate the excellent performance of the proposed model in this paper.

## 5. Conclusions

Lung cancer is one of the most lethal malignant tumors globally, characterized by heterogeneous histological subtypes and significant variability in treatment management. Accurate detection and classification of different cancer subtypes play a crucial role in selecting the best treatment strategies. This paper proposes an improved lung cancer detection network based on YOLO V8, which enhances the focus on lung cancer information in the Backbone by introducing large kernel separable attention machines after all C2f layers, and strengthens the association of information at different scales in the Neck. Additionally, by integrating depth-wise convolution and Coordinate Attention mechanisms into the SPPF, the network better retains lung cancer information in deep features. In the regression branch, MPDIOU Loss is used to enhance the association between the predicted and labeled bounding boxes, better capturing the shape and position of the target, thereby improving the model’s performance in detecting small targets. Through a series of experimental validations, the proposed network surpasses other mainstream detection networks in terms of mean average precision and accuracy. Furthermore, the experimental results are graphically presented, intuitively showing the model’s sensitivity in recognizing different lung cancer subtypes.

However, the current model has been primarily tested on small sample datasets, and its robustness and generalization capabilities have not been fully validated on large-scale datasets. Additionally, the model is still in the theoretical experimental stage and has not been applied in actual medical environments, so its timeliness and effectiveness in practical applications need further evaluation.

To address these issues, future research should focus on the following aspects:

Optimizing datasets and network structures: Construct and annotate large-scale cancer image datasets and adjust the network structure to improve detection performance.

Exploring multimodal data fusion: Integrate various data modes, such as medical imaging, gene sequencing, and pathological slices, to provide more comprehensive cancer classification information.

Combining clinical practice and feedback: Collaborate with medical professionals to understand practical needs, optimize the network to meet clinical application requirements, and collect feedback for continuous model improvement.

## Figures and Tables

**Table 1:** The pseudocode of the training process.

Algorithm YOLO V8-LCM
Input: input CT images and their annotations.
Output: Model weights and detection indicators.
for *i*th-fold, (train-dataset, val-dataset) in K-fold dataset:
for *j*th-epoch in range(epochs):
for step, train-data in enumerate (train-dataset):
Using train-data to train weights using YOLO V8-LCM model in Section 3.
Calculate the classification loss and MPDIOU Loss and return the gradient.
Update parameters in the network in this step.
end
Save the weights of the optimal model in this jth-epoch.
end
Verify on the val-dataset and calculate detection indicators in this ith-fold.
end
Calculate the mean and standard deviation of the detection indicators in the K-fold dataset.

**Table 2:** Analysis of experimental results (average ± standard deviation).

Classess	Precision	Recall	mAP (50%)	mAP (50–90%)
ALL-LESIONS	0.954 ± 0.0028	0.968 ± 0.0037	0.981 ± 0.0052	0.629 ± 0.0152
Class 0 (SCLC)	0.963 ± 0.0023	0.958 ± 0.0029	0.986 ± 0.0037	0.619 ± 0.0131
Class 1 (LCC)	0.961 ± 0.0025	0.947 ± 0.0032	0.981 ± 0.0028	0.592 ± 0.0093
Class 2 (ADC)	0.952 ± 0.0031	0.951 ± 0.0026	0.966 ± 0.0029	0.658 ± 0.0142
Class 3 (SCC)	0.967 ± 0.0027	0.979 ± 0.0028	0.986 ± 0.0033	0.663 ± 0.0152

**Table 3:** Ablation experiments (average ± standard deviation).

Module	Precision	Recall	Specificity	mAP (50%)	mAP (50–95%)
YOLO V8	0.942 ± 0.0047	0.953 ± 0.0073	0.958 ± 0.0089	0.968 ± 0.0061	0.589 ± 0.0173
YOLO V8-CM	0.945 ± 0.0036	0.956 ± 0.0057	0.962 ± 0.0062	0.972 ± 0.0058	0.617 ± 0.0147
YOLO V8-LM	0.949 ± 0.0042	0.967 ± 0.0053	0.96 ± 0.0059	0.976 ± 0.0051	0.619 ± 0.0136
YOLO V8-LC	0.951 ± 0.0031	0.963 ± 0.0066	0.961 ± 0.0057	0.97 ± 0.0055	0.6138 ± 0.0143
YOLO V8-LCM	0.954 ± 0.0028	0.968 ± 0.0052	0.969 ± 0.0061	0.981 ± 0.0052	0.629 ± 0.0152

**Table 4:** Detection of comparison experiment in test dataset (average ± standard deviation).

Model	Precision	Recall	Specificity	mAP (50%)	Parameters	Speed
SSD	0.896 ± 0.013	0.933 ± 0.013	0.922 ± 0.019	0.939 ± 0.0168	134 M	5.3 ms
Faster R-CNN	0.907 ± 0.018	0.926 ± 0.018	0.923 ± 0.021	0.957 ± 0.013	430 M	12.2 ms
YOLO V3	0.918 ± 0.0087	0.958 ± 0.012	0.955 ± 0.083	0.965 ± 0.0098	32 M	4.5 ms
YOLO V5	0.925 ± 0.0066	0.956 ± 0.0066	0.961 ± 0.0068	0.973 ± 0.0068	1.8 M	2.9 ms
YOLO V7	0.919 ± 0.0067	0.947 ± 0.0078	0.956 ± 0.0078	0.965 ± 0.0072	6.03 M	3.8 ms
YOLO V8	0.922 ± 0.0051	0.951 ± 0.0069	0.955 ± 0.0086	0.969 ± 0.0077	3.1 M	3.3 ms
YOLO V8-LCM	0.934 ± 0.0035	0.958 ± 0.0058	0.966 ± 0.0065	0.977 ± 0.0065	3.2 M	3.6 ms

**Table 5:** Accuracy comparison experiment in different references.

References	Data Type	Database	Subtypes of Lung Cancer	Samples	Algorithm Model	Results
Saad et al. [31]	CT	Lung1	ADC, SCC, LCC	317	SVM	ACC = 0.78
Liu et al. [32]	CT	Lung1	ADC, SCC, LCC, NOS	349	SVM	ACC = 0.86
Wang et al. [33]	CT	Private dataset	SCC, SQC, IA, ISA	168	ResNet	ACC = 0.86
Pang et al. [34]	CT	Private dataset	ADC, SCC, SQC	1183	DenseNet	ACC = 0.90
Han et al. [35]	CT	Private dataset	ADC, SQC	283	VGG16	ACC = 0.84
Gao et al. [36]	CT	Lung1	SCC, LCC	169	GBRT	ACC = 0.96
Jacob et al. [37]	PET/CT	Lung-PET-CT-Dx	ADC, SCC, SQC	204	Shadow CNN	ACC = 0.95
Wang et al. [32]	PET/CT	Lung-PET-CT-Dx	ADC, SCC, SQC	232	ResNet	ACC = 0.81
Marentakis et al. [38]	CT	TCIA	ADC, SCC	914	LSTM+ Inception	AUC = 0.74
Kosaraju et al. [39]	WSI	GNUH	SCC, ADC, SCLC, LCNEC	40,000 patches	CAT-Net	Micro F1 = 0.701
Khalil et al. [40]	PET/CT	Lung-PET-CT-Dx	ADC, SCC, SQC	270	MobiNet	ACC = 0.74
Khalil et al. [40]	PET/CT	Lung-PET-CT-Dx	ADC, SCC, SQC	270	Xception	ACC = 0.78
Khalil et al. [40]	PET/CT	Lung-PET-CT-Dx	ADC, SCC, SQC	270	DETR	ACC = 0.96
Our approach	CT	Lung-PET-CT-Dx, NSCLC-Radiomics, NSCLC-Radiogenomics	ADC, SQC, SCC, LCC	814	YOLO V8-LCM	ACC = 0.961

## Data Availability

No new data were created or analyzed in this study. Data sharing is not applicable to this article.
